# Preoperative myocardial strain, mesenteric resistance, and aortopulmonary collateral flow are associated with pleural effusion duration after fontan completion

**DOI:** 10.3389/fped.2026.1787018

**Published:** 2026-05-29

**Authors:** Z. Alsafi, F. Dangardt, P. Frieberg, C. De Lange, K. Hanséus, M. Synnergren, K. Tran, M. Odermarsky, M. Carlsson, P. Liuba

**Affiliations:** 1 Pediatric Cardiology, Childreńs Heart Centre, Skåne University Hospital, Lund, Sweden; 2 Pediatrics, Department of Clinical Sciences Lund, Lund University, Lund, Sweden; 3 Pediatric Heart Centre, The Queen Silvia Childreńs Hospital, Sahlgrenska University Hospital, Region Västra Götaland, Gothenburg, Sweden; 4 Department of Molecular and Clinical Medicine, Institue of Medicine, Sahlgrenska Academy, University of Gothenburg, Gothenburg, Sweden; 5 Clinical Physiology, Department of Clinical Sciences, Lund University, Lund, Sweden; 6 Department of Clinical Physiology, Skåne University Hospital, Lund, Sweden; 7 Department of Pediatric Radiology, Queen Silvia Childreńs Hospital, Sahlgrenska University Hospital, Region Västra Götaland, Gothenburg, Sweden; 8 Institute of Clinical Sciences, Sahlgrenska Academy, University of Gothenburg, Gothenburg, Sweden; 9 Department of Clinical Physiology, Karolinska University Hospital, Stockholm, Sweden

**Keywords:** aortopulmonary collateral, cardiac magnet resonance imaging (CMR), global longitudinal strain (GLS), mesenteric perfusion, pleural effusion, single ventricle (SV), total cavopulmonary connection (TCPC)

## Abstract

**Introduction:**

The contribution of aortopulmonary collateral (APC) flow to pleural effusion (PE) after total cavopulmonary connection (TCPC) remains unclear. Excessive APC flow may alter mesenteric perfusion and increase ventricular volume load. We evaluated echocardiographic and mesenteric flow parameters as predictors of PE duration and their association with magnetic resonance imaging (MRI)-derived APC flow.

**Methods:**

We prospectively studied patients with single ventricle (SV) physiology referred for TCPC at the Children's Heart Centers in Lund and Gothenburg, the two referral centers for pediatric cardiac surgery in Sweden. Preoperative assessment included 2D longitudinal strain from four-chamber (4CH) view and 3D global longitudinal strain (GLS), Doppler derived resistance index (RI) in the superior mesenteric artery (SMA) and cardiac MRI. APC flow was quantified using two MRI-based methods: (A) pulmonary venous minus arterial flow, and (B) aortic flow minus total caval flow, both indexed to body surface area.

**Results:**

Thirty-four patients (median age at TCPC 2.8 (range:1.8–8.2) years; female: 44%; dominant right ventricle: 56%) were included. PE duration was significantly associated with higher 4CH-longitudinal strain (*r* = 0.34, *p* = 0.045). Pre-TCPC SMA RI was not significantly associated with PE duration but was correlated with APC flow. Although 3D GLS was not directly associated with PE duration, it correlated with APC flow quantified by both MRI methods (method A: *r* = 0.58, *p* = 0.006; method B: *r* = 0.48, *p* = 0.03). APC flow calculated using method B, and lower IVC flow (*r* = 0.73, *p* < 0.001) were associated with longer PE (*r* = 0.53, *p* = 0.004).

**Discussion:**

Prolonged pleural effusion after TCPC is associated with APC burden, echocardiographic markers of ventricular volume loading, and impaired mesenteric perfusion. GLS and SMA resistance index may reflect APC-related hemodynamic disturbance and serve as accessible, non-invasive surrogates to help identify patients at risk for adverse early TCPC outcomes.

PE duration after TCPC is associated with echocardiographic markers of volume overload and impaired mesenteric perfusion. GLS and SMA RI may serve as non-invasive surrogates for APC burden measured via MRI.

## Introduction

Pleural effusion (PE) remains a frequent and clinically significant complication following total cavopulmonary connection (TCPC) in patients with single-ventricle (SV) physiology, often leading to extended hospitalization and increased morbidity (1, 2). Early identification of patients at risk could improve perioperative management and outcomes. Aortopulmonary collaterals (APCs), which commonly develop after the Glenn and persist following TCPC (3–6) have been proposed as contributors to PE (7, 8) by increasing pulmonary blood flow and pressure, imposing additional volume load on the systemic ventricle, and competing with systemic venous return (5–11). However, published studies have reported conflicting results regarding this association, which may be partly due to differences in how APC flow is quantified (5, 7, 10, 12–16).

Cardiac magnetic resonance imaging (MRI) remains the reference standard for measuring APC flow, but its use is limited by availability, cost, and the need for sedation or anesthesia (14). Echocardiography, in contrast, is widely accessible, repeatable, and can provide valuable indirect information on APC burden, ventricular loading conditions, and systemic flow patterns. Parameters such as ventricular strain and indices of mesenteric blood flow have been used to characterize systemic and splanchnic perfusion in single-ventricle circulation and may provide indirect insight into the hemodynamic burden associated with excessive APC flow (17–21). However, few studies have systematically evaluated these echocardiographic markers in relation to quantified APC burden, and their potential as reliable surrogates remains underexplored. Further, it is unclear how well these non-invasive measures predict clinically significant outcomes such as PE duration.

This prospective study aimed to identify echocardiographic variables associated with postoperative PE following TCPC and to examine their relationship with APC flow measured via MRI. Establishing echocardiographic surrogates for APC burden could provide a widely available tool for early risk stratification and postoperative management in this vulnerable patient population.

## Methods

### Study population

This prospective study was conducted at the two referral centers for pediatric cardiac surgery in Sweden, Lund and Gothenburg University Hospitals, from 2019 to 2025, where patients were followed from hospital admission for TCPC until postoperative discharge. All single ventricle (SV) patients scheduled for TCPC surgery were invited to participate in the study. Families were contacted prior to or during the admission for TCPC surgery when a written informed consent was obtained. No patients in the study cohort underwent transcatheter embolization of APCs between the preoperative MRI examination and TCPC surgery.

### Inclusion and exclusion criteria

Patients with comorbidities beyond SV physiology, such as 22q11 microdeletion syndrome or trisomy 21, were excluded. Patients were also excluded if MRI studies were incomplete due to awakening from sedation, or if they had a baseline low heart rate, given the additional bradycardic effect of nasal dexmedetomidine that was given for sedation. Additionally, patients with non-MRI compatible implants were excluded.

### Preoperative imaging and sedation procedure

As an addition to the routine preoperative management, patients underwent cardiac MRI one to two days before surgery. Mild sedation was achieved with intranasal dexmedetomidine, administered at a dose of 2–3 mg/kg, followed by an additional dose of 1–1.5 mg/kg approximately 40 min later, just before the MRI scan. From the initial administration until the child awakened after the scan, continuous monitoring of the heart rate and saturation was performed using ECG and pulse oximetry, respectively. Blood pressure was measured before sedation and then automatically monitored throughout the scan and post-sedation until the child fully awakened. During this sedation, an echocardiographic examination was also performed. An ultrasound of the mesenteric arteries was also conducted at the same time and was later repeated postoperatively before discharge. All MRI scans were performed in the afternoon after the patients were fed and during their usual afternoon rest. A parent or a nurse stayed with the patient during the examination to provide comfort if needed.

### Definition of postoperative outcome variables

PE duration was defined as the number of days from surgery until the chest or mediastinal tube was removed and not re-inserted. Chest tube removal was performed according to institutional clinical practice based on drainage volume, radiographic findings and clinical status rather than a strict standardized protocol.

### Ethics approval and informed consent

The study was approved by the Swedish Ethical Review Authority in accordance with national regulations for clinical research (Ethical Approval Numbers: 2009/616, 2017/522, 2017/898 and 2016-445-31M), and written informed consent was obtained from the parents of all participants.

### Imaging protocol

#### A. Echocardiography

All patients underwent a preoperative transthoracic echocardiographic examination conducted by an experienced echocardiographer. Images were acquired using appropriate transducers based on the patient's weight and age, and all examinations were performed on clinically available machines (Phillips EPIQ Elite and GE Vivid E95). Raw DICOM (Digital Imaging and Communications in Medicine) imaging data were digitally stored and both 2D and 3D analyses were carried out and analyzed offline, by one single experienced observer at a dedicated workstation. Intra-observer variability was assessed by having the same observer repeat ten measurements of GLS using both 2D and 3D echocardiography, as well as ejection fraction (EF) using 3D echocardiography, with a minimum interval of two weeks between each measurement. An inter-observer variability analysis was also conducted by having another experienced echocardiographer repeat ten measurements of GLS using 2D echocardiography. The 2D echo analysis was performed offline using Intellispace Cardiovascular (ISCV), while 3D and strain analyses were conducted using TomTec Arena 2.53 (2024 Philips Medical systems, The Netherlands). For 2D global longitudinal strain (GLS), automated software for left ventricular speckle tracking was used, and for 3D analysis, the 4D LV-analysis software was applied.

##### 2D Echocardiography—strain analysis

**Figure 1:**
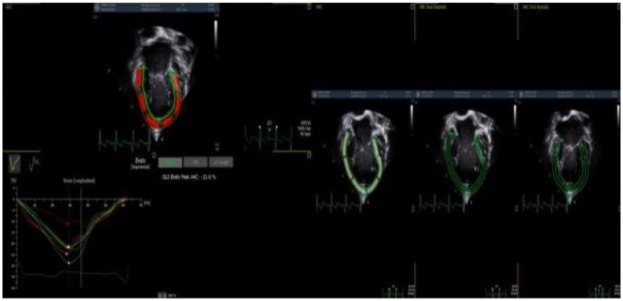
Global longitudinal strain (*G*LS) measured from an apical four-chamber view, tracing the endocardial border. Peak systolic longitudinal strain is recorded from all six segments of the myocardium and GLS is calculated as the average of all available segments.

From a four-chamber view, 2D images optimized for speckle tracking were acquired with frame rates (median 76, range 30–117 frames/s). Using automated software for left ventricular (LV) speckle tracking, the endocardial border was automatically traced and manually adjusted as needed. The software divided the systemic ventricle into six segments, with segments excluded if the myocardium was not adequately visualized for speckle tracking. Peak systolic longitudinal strain, defined as the percent change in segment length from end-diastole, was recorded for each segment. GLS was calculated as the average of all available regional segments (Figure 1). Patients with suboptimal acoustic windows for 2D speckle tracking were excluded from the analysis. As only the apical four-chamber view was used for 2D strain analysis, this parameter is referred to as four-chamber (4CH) longitudinal strain rather than GLS.

##### 3D Echocardiography—volume measurements and strain analysis

**Figure 2:**
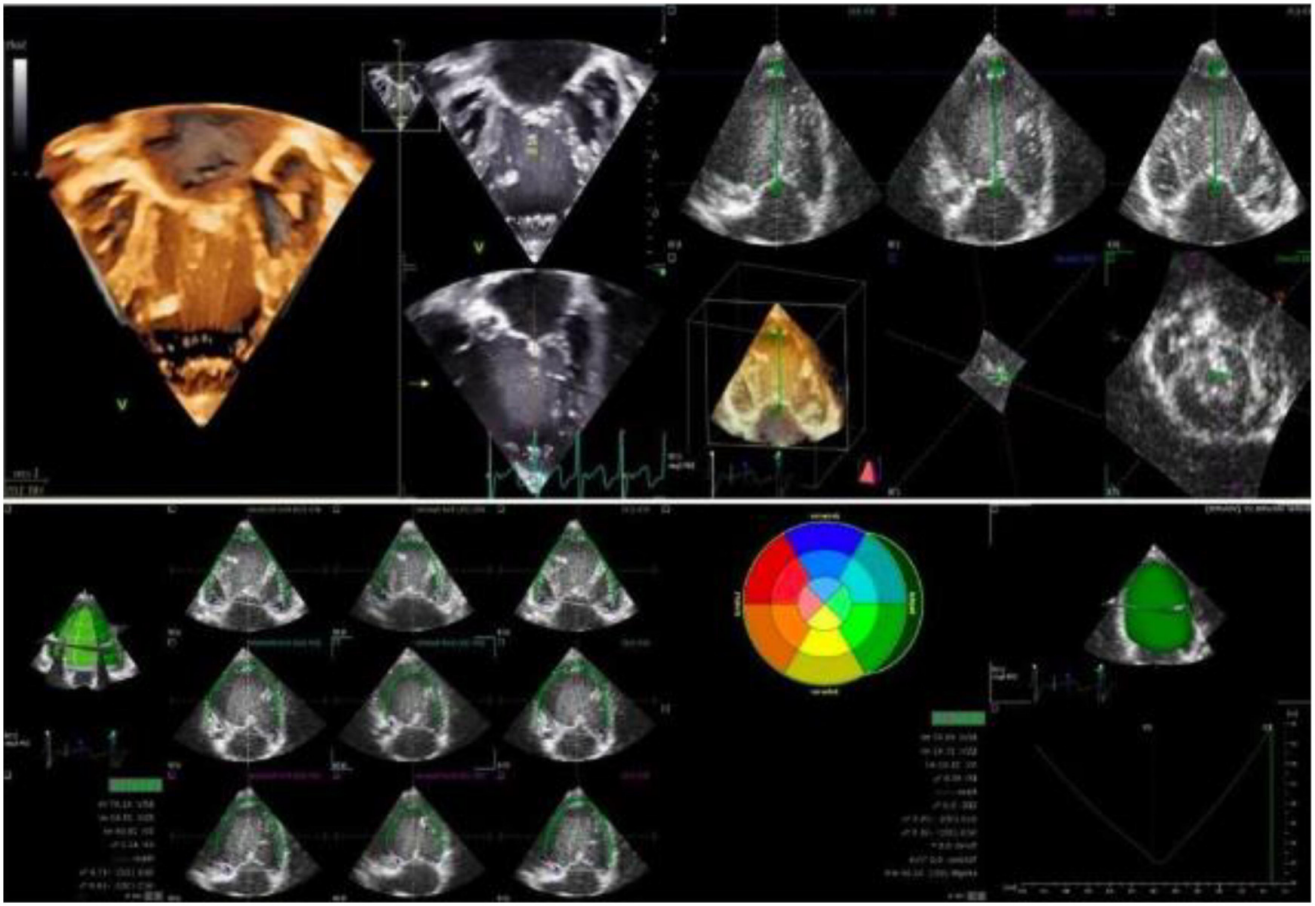
A full 3-dimensional volume is captured from a apical four-chamber view. The atrioventricular valve, apex and aorta/neoaorta is identified and the endocardial border is traced to calculate volumes, ejection fraction and strains.

The 3D full-volume echocardiogram was acquired from an apical position, with the sector covering the entire systemic ventricle, including the apex. The 3D dataset consisted of 2–4 sub-volumes, ECG-gated at the highest achievable frame rate (median 35, range 13–54 frames/s). Multiple datasets were obtained for each patient, and the best-quality images, free of stitch artifacts, were selected. In patients where the required frame rate, image quality could not be achieved, and examination with detectable stitch artifacts were excluded from the analysis. For analysis, the systemic ventricle long-axis was manually aligned from the center of the atrioventricular (AV) valve to the apex across three apical views (4-chamber, 3-chamber, and 2-chamber). The reference point for the aortic valve was placed at the aortic or neo-aortic valve position. The software automatically identified the endocardial border, which was manually adjusted as needed, and tracked the endocardial border throughout the cardiac cycle. Automatic calculations for end-diastolic volume (EDV), end-systolic volume (ESV), EF, and several strains were generated. However, only GLS was used in this study (Figure 2). In patients with a morphologically right systemic ventricle, ventricular volumes and EF were obtained using the same three-dimensional dataset and analyzed with the TomTec 4D LV-Analysis software. Although originally designed for left ventricular analysis, the software has been previously applied to systemic right ventricles by adapting endocardial contouring and manually adjusting the borders when necessary to ensure accurate tracking of the ventricular cavity (22, 23).

##### Ultrasound of the superior mesenteric and celiac arteries

**Figure 3:**
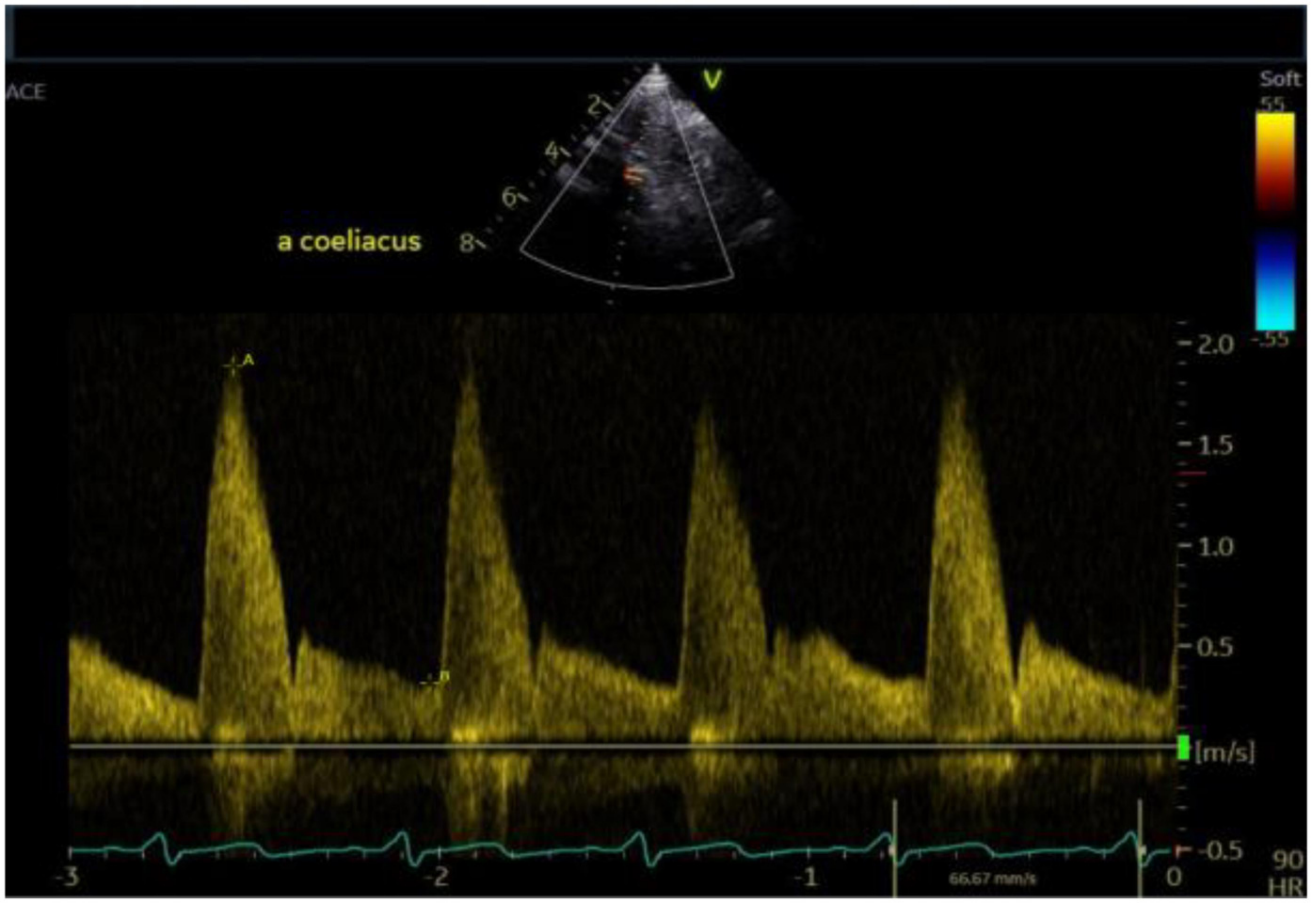
The blood flow of the mesenteric arteries are captured with e pulsed-wave Doppler from a sagittal subcostal view. Peak systolic velocity **(A)** and end-diastolic velocity **(B)** is measured, and resistance index is calculated.

Data acquisition was performed using a low-wall filter from a sagittal subcostal view, with the probe positioned to minimize the angle of interrogation of the superior mesenteric and celiac arteries (CA). Measurements were taken from the proximal portions of the arteries to avoid including blood flow from the abdominal aorta at the arterial origins. Three Doppler waveforms were recorded per patient. Peak systolic velocity (s) and end-diastolic velocity (d) were measured and averaged across the three measurements (Figure 3). The arterial resistance index (RI) was calculated using the following equation:

$$\mathrm{RI}=\frac{\mathrm{peak}\;\mathrm{systolic}\;\mathrm{velocity}\;\text{–}\;\mathrm{end}\text{-}\mathrm{diastolic}\;\mathrm{velocity}}{\mathrm{peak}\;\mathrm{systolic}\;\mathrm{velocity}}$$

End-diastole was defined as the lowest point on the Doppler waveform.

#### B. Magnetic resonance imaging

MRI cine images and flow measurements of the heart and intrathoracic vessels were acquired in Lund University hospital, using a 1.5T Siemens Aera scanner (Siemens Healthineers, Erlangen, Germany) and in Gothenburg University hospital, using a 3T scanner Signa Architect (GE HealthCare, Chicago, IL, USA). Two-dimensional phase contrast flow measurements were performed in Lund and 4D flow measurements in Gothenburg, analyzing the left and right pulmonary arteries (LPA, RPA), the left and right pulmonary veins (LPV, RPV), the superior and inferior vena cava (SVC, IVC), and the aorta (Ao). Stroke volume was derived from flow measurements in the ascending aorta and indexed to body surface area (BSA) to obtain the cardiac index (CI). All other flow measurements were also indexed for BSA. One experienced reader performed all MRI segmentation and analysis, using the freely available software *Segment* (Medviso AB, Lund, Sweden; https://medviso.com/segment) (24). Analysis was performed after each completed MRI-scan; therefore, it was not blinded and no analysis of variability was conducted.

To calculate the EF of the systemic ventricle, endo- and epicardial contours were manually traced at end-diastole and end-systole. The papillary muscles and trabeculae were excluded from the lumen but included in the ventricular volume calculations. The software automatically calculated the ventricular EF, EDV, and stroke volume.

Aortopulmonary collateral flow was determined using the following methods:

- **Method A**: The difference between pulmonary venous (PV) flow and pulmonary artery (PA) flow.
- **Method B**: The difference between aortic (Ao) flow and total caval (SVC + IVC) flow. Both methods were indexed for BSA.

The IVC% was calculated as the percentage of IVC flow relative to the total caval flow (SVC + IVC).

### Statistical analysis

Statistical analysis was performed using SPSS Version 28.0.0.0 (190) for Mac (SPSS, Inc., Chicago, IL, USA). All parameters are reported as median and range, while proportions are expressed as percentages. Linear regression analysis, using Spearman's correlation coefficient, was applied to examine the relationship between MRI and echo variables and PE drainage duration. The same method was used to correlate echo and MRI variables, specifically to assess the relationship between APC-flow and IVC%. PE duration, IVC% and GLS by 3D echocardiography were log-transformed for visualization purposes in regression plots to reduce skewness; however, correlations were assessed using Spearman's rank correlation, which does not assume normal distribution. A paired *t*-test was applied to evaluate differences in blood flow and RI in the superior mesenteric and celiac arteries. A *p*-value of <0.05 was considered statistically significant. Given the relatively small sample size and varying availability of imaging datasets, multivariable regression analyses were not performed to avoid model overfitting. Instead, exploratory univariable correlation analyses were conducted. Missing data were handled using available-case analysis, whereby each statistical test included all patients with complete data for the variables analyzed. No imputation of missing values was performed.

An intra-observer variability analysis using the intraclass correlation coefficient (ICC) was performed for EF measured by 3D echocardiography, as well as 4CH-longitudinal strain assessed by 2D and GLS by 3D echocardiography. An inter-observer variability analysis was also performed using ICC for 4CH-longitudinal strain by 2D echocardiography.

## Results

### Patient characteristics and data exclusion

A total of 36 patients were included (19 from Lund and 17 from Gothenburg). Two patients were excluded due to awakening from sedation during the MRI scan. Another five MRI scans were excluded during analysis due to poor image quality affecting flow and volume quantifications. In the echocardiographic examinations, four patients were excluded from the 2D strain analysis as well as the mesenteric and celiac artery flow measurements, due to poor image quality. Furthermore, twelve 3D-echocardiographic examinations were excluded due to poor image quality.

The median age at the time of TCPC was 2.8 years (range 1.8–8.2). Among the patients, 44% were female, and 56% had a morphologically dominant right ventricle. The median duration of postoperative PE drainage was 12.5 days (range 4.0–101.0), and the median hospital LOS was 22.0 days (range 13.0–107.0). Demographic details are presented in Table 1. Echo and MRI-data are presented in Tables 2, 3.

**Table 1:** Patient demographic data.

Pre-TCPC	*n* = 34
Age (years)	2.8 (1.8–8.20)
Glenn-TCPC interval (years)	2.3 (0–7.7)
Male, *n* (%)	19 (56)
Diagnosis, RV *n* (%)	19 (56)
Tricuspid atresia	5 (15)
Double inlet left ventricle	2 (5)
Pulmonary atresia	5 (15)
Hypoplastic left heart syndrome	9 (26)
Double outlet right ventricle	3 (9)
Atrioventricular septal defect with LV dominance	1 (3)
Atrioventricular septal defect with RV dominance	6 (18)
Other^a^	3 (9)
Weight (kg)	12.8 (11.1–27.0)
Length (cm)	90.0 (80.0–129.0)
BSA, m^2^	0.56 (0.49–0.98)
SpO2, %	83 (75–91)
Cardiopulmonary bypass (min)	98.5 (43.0–254.0)
PICU (days)	2.5 (1–30)
Pleural effusion days	13.0 (4–101)
LOS (days)	22.0 (13–107)

a Other includes 1 ccTGA with right ventricle dominance, 1 ccTGA with left ventricle dominance and 1 Ebstein's anomaly.

TCPC, Total cavopulmonary connection, RV, Right ventricle, BSA, Body surface area, PICU, Pediatric intensive care unit, LOS, length of hospital stay. Data are expressed as median and range.

**Table 2:** Echocardiographic data.

Parameter	Median (range)
Echocardiography data pre-TCPC	
3D—End diastolic volume (mL) (*n* = 22)	60.5 (18.7–99.9)
3D—Ejection fraction (%) (*n* = 22)	49.7 (40.1–57.7)
3D Global longitudinal strain (%) (*n* = 21)	−23.1 [(−10.3)–(−31.9)]
2D Global longitudinal strain (%) (*n* = 31)	−18.7 [(−13.6)–(−25.8)]
Superior mesenterica artery flow in systole (cm/s) (*n* = 31)	120.0 (57.7–172.2)
Superior mesenterica artery flow in diastole (cm/s) (*n* = 31)	8.7 (0.0–29.5)
Superior mesenteric artery Resistance index (*n* = 31)	0.93 (0.78–1.0)
Celiac artery flow in systole (cm/s) (*n* = 31)	129.2 (64.3–258.9)
Celiac artery flow in diastole (cm/s) (*n* = 31)	20.9 (0.0–54.2)
Celiac artery Resistance index (*n* = 31)	0.83 (0.75–1.0)
Echocardiography data post-TCPC	
Mesenterical flow in systole (cm/s) (*n* = 29)	86.7 (50.4–211.4)
Mesenterical flow in diastole (cm/s) (*n* = 29)	13.0 (5.2–25.7)
Mesenterical Resistance index (*n* = 29)	0.87 (0.73–0.96)
Celiac flow in systole (cm/s) (*n* = 29)	99.9 (64.0–212.2)
Celiac flow in diastole (cm/s) (*n* = 29)	21.3 (7.7–39.8)
Celiac Resistance index (*n* = 29)	0.81 (0.73–0.92)

Data are expressed as median and range. *3D,* three-dimensional, *2D,* two-dimensional.

**Table 3:** Magnetic resonance imaging data.

Parameter	Median (range)
MRI data pre-TCPC	
End diastolic volume (mL/m^2^) (*n* = 29)	101.6 (58.7–190.8)
Ejection fraction (%) (*n* = 29)	49.0 (32.0–63.0)
Atrioventricular valve regurgitation (%) (*n* = 27)	6.7 (0.0–44.1)
Cardiac index (L/min/m^2^) (*n* = 29)	3.7 (2.2–7.3)
Total pulmonary artery flow (L/min/m^2^) (*n* = 29)	1.5 (0.97–121.0)
Left pulmonary artery flow, relative to total pulmonary arterial flow (%) (*n* = 29)	42.2 (14.9–67.0)
Total pulmonary venous flow (L/min/m^2^) (*n* = 29)	2.2 (1.6–5.0)
Total APC blood flow (Method A) (L/min/m^2^) (*n* = 29)	0.91 (0.18–2.46)
Total APC blood flow (Method B) (L/min/m^2^) (*n* = 29)	0.91 (0.0–1.92)
Superior vena cava flow (L/min/m^2^) (*n* = 29)	1.4 (0.42–1.84)
Inferior vena cava flow (L/min/m^2^) (*n* = 28)	1.3 (0.38–2.55)
Inferior vena cava flow, relative to total caval blood flow (%) (*n* = 28)	47.0 (30.6–60.0)

Data are expressed as median and range. APC, Aortopulmonary collaterals.

### Associations between imaging parameters and pleural effusion duration

**Figure 4:**
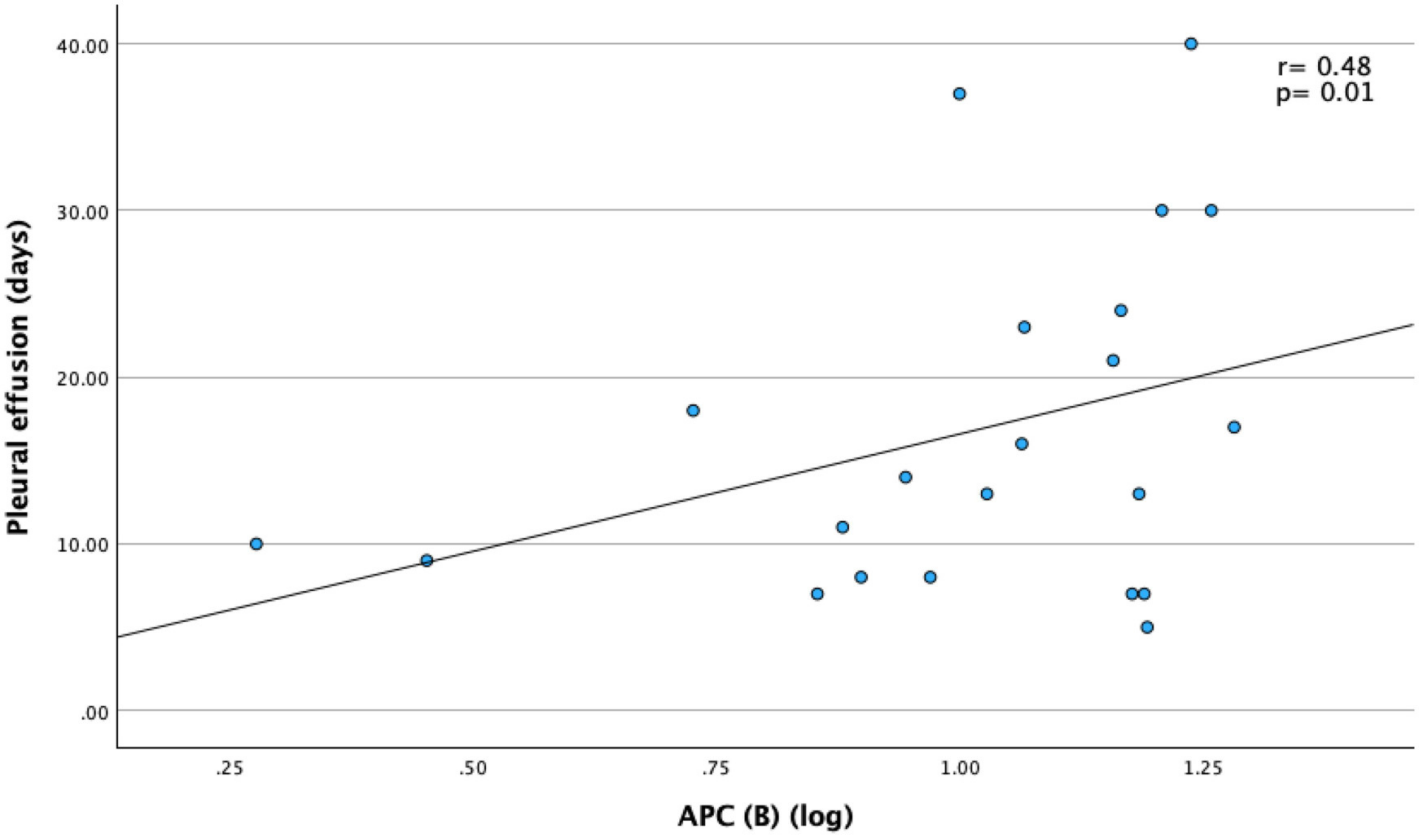
Linear regression illustrating the association between postoperative pleural effusion duration and log-transformed aortopulmonary collateral flow, calculated using method B as [Ao-(SVC + IVC)].

**Figure 5:**
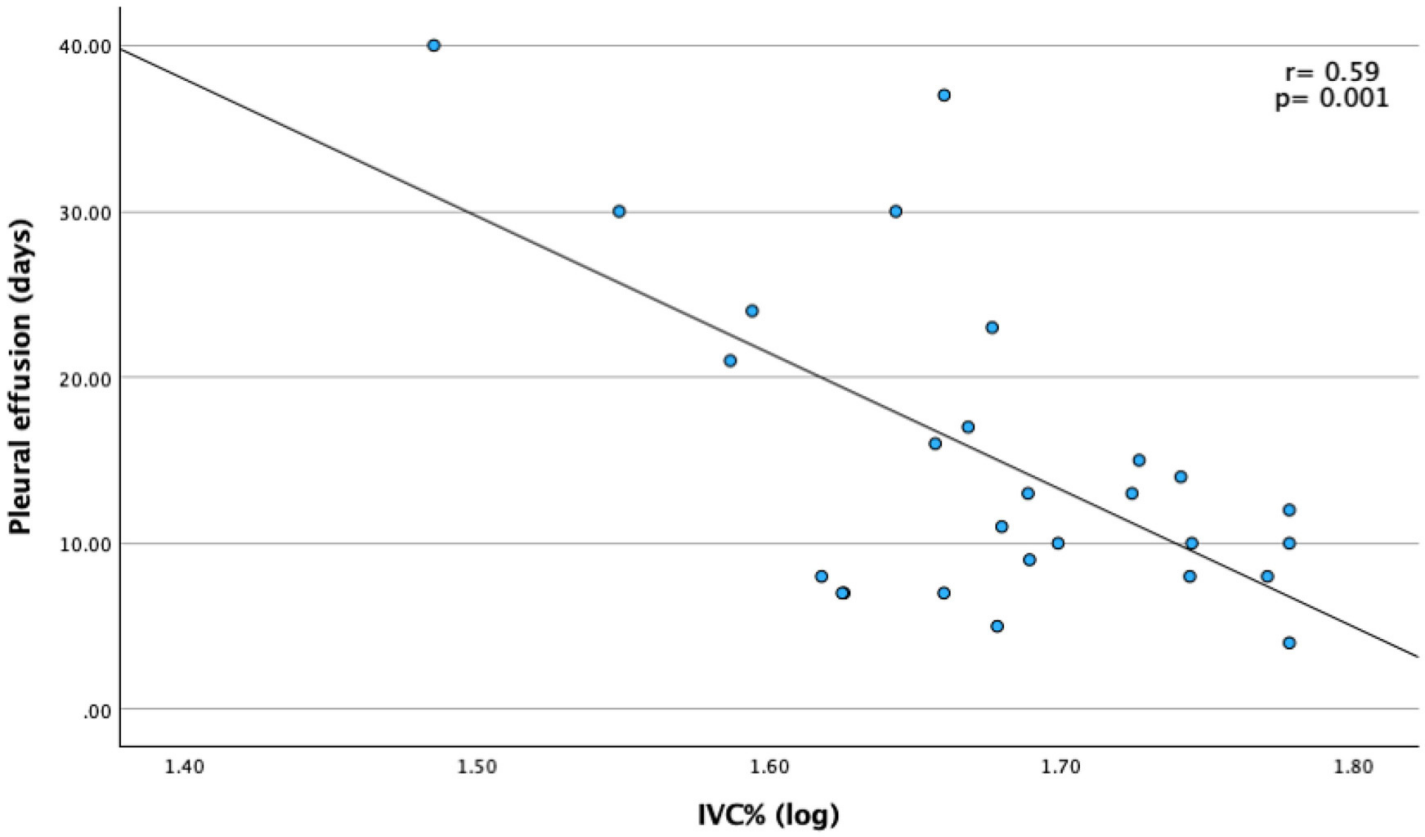
Linear regression illustrating the association between postoperative pleural effusion duration and inferior vena cava flow expressed relative to total caval flow [IVC/(SVC + IVC)].

**Figure 6:**
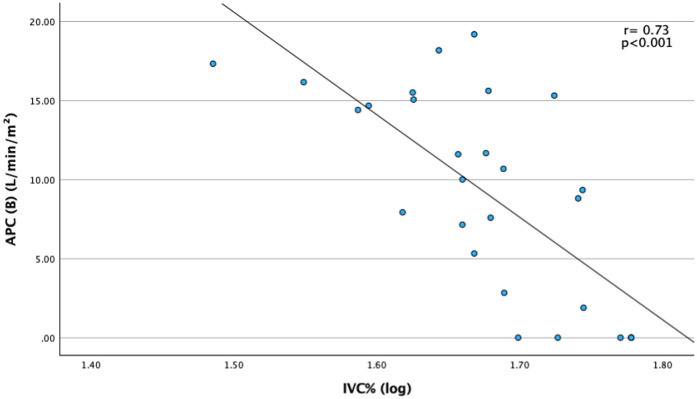
Linear regression illustrating the association between APC-flow calculated using method B [Ao-(SVC + IVC)] and inferior vena cava flow expressed relative to total caval flow [IVC/(SVC + IVC)].

Postoperative PE drainage duration was positively correlated with a higher amount of APC flow when calculated using method B (*r* = 0.48, *p* = 0.01) (Figure 4). A lower IVC% was associated with longer PE drainage duration (*r* = 0.5, *p* = 0.001) (Figure 5) and was inversely correlated with APC flow (*r* = 0.73, *p* < 0.001) (Figure 6). The correlation between IVC% and APC flow was not statistically significant when APC flow was calculated using method A. Although method A showed a similar trend, the correlation with IVC% did not reach statistical significance (*p* = 0.06).

Higher cardiac index (CI) (*r* = 0.37, *p* = 0.03) and higher 4CH-longitudinal strain measured by 2D echocardiography (*r* = 0.34, *p* = 0.045) were also associated with longer PE duration. No significant associations were found between PE duration and age at TCPC, systemic right ventricle and those with a systemic left ventricle, cardiopulmonary bypass time, EDV or EF% by MRI, or EF% and GLS by 3D echocardiography (all *p* > 0.1) (Table 4).

**Table 4:** Correlations between pleural effusion duration and MRI- and echocardiographic variables.

Parameter	*p*-value	r
PE—age at TCPC (*n* = 34)	0.509	0.12
PE—systemic ventricle (*n* = 34)	0.918	0.02
PE—cardiopulmonary bypass time (*n* = 32)	0.230	0.22
PE—EDV (MRI) (*n* = 29)	0.851	0.04
PE—EF% (MRI) (*n* = 29)	0.309	0.20
PE—EF% (3D echo) (*n* = 21)	0.762	0.07
PE—GLS (3D echo) (*n* = 21)	0.426	0.19
PE—AV regurgitation (*n* = 27)	0.221	0.24

PE, pleural effusion, TCPC, Total cavopulmonary connection, EDV, end-diastolic volume, MRI, magnetic resonance imaging, EF, ejection fraction, 3D echo, three-dimensional echocardiography, AV, atrioventricular valve.

**Figure 7:**
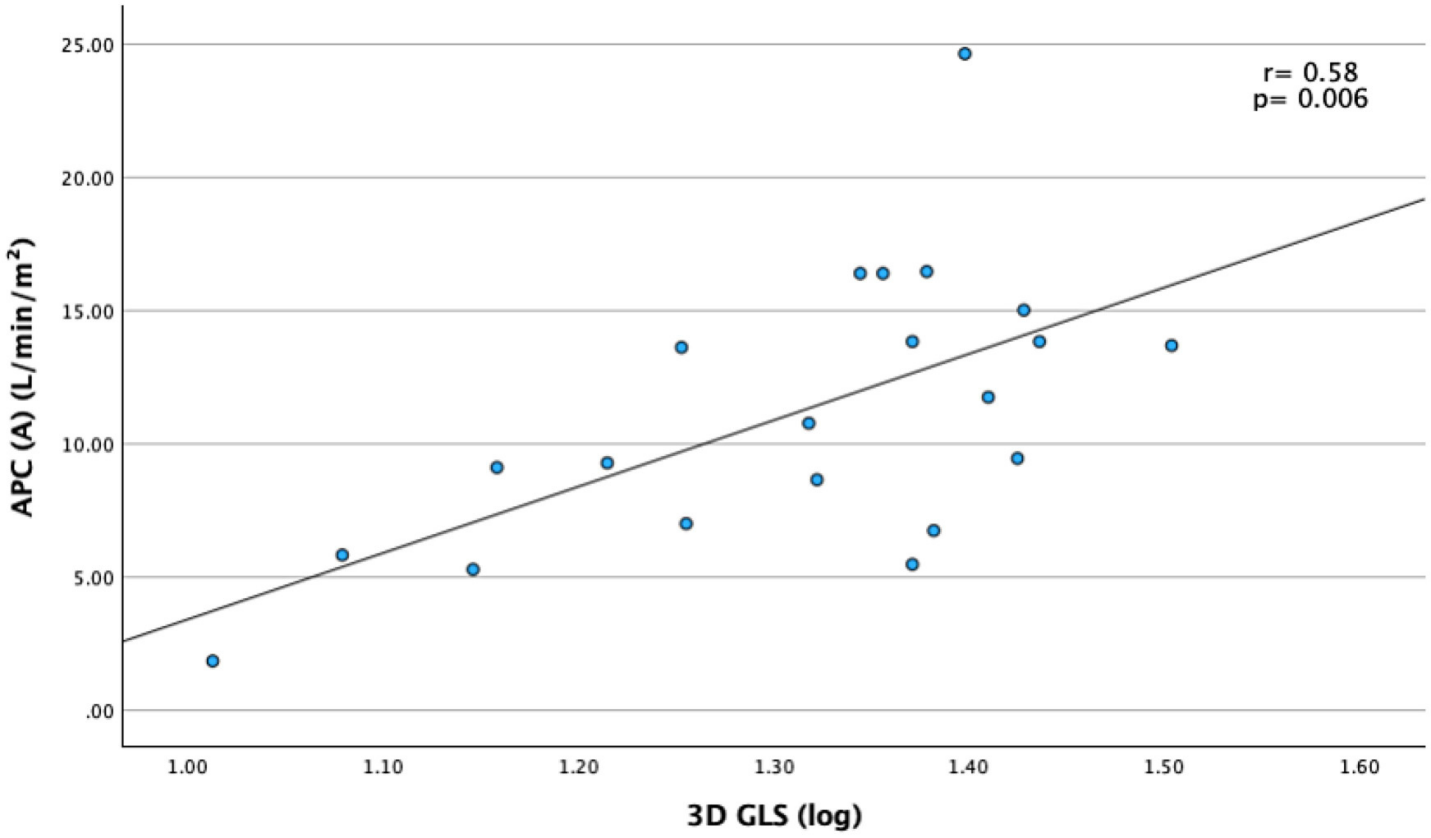
Linear regression illustrating the association between APC-flow calculated using method A (PV-PA) and log-transformed global longitudinal strain measured by 3D echocardiography.

**Figure 8:**
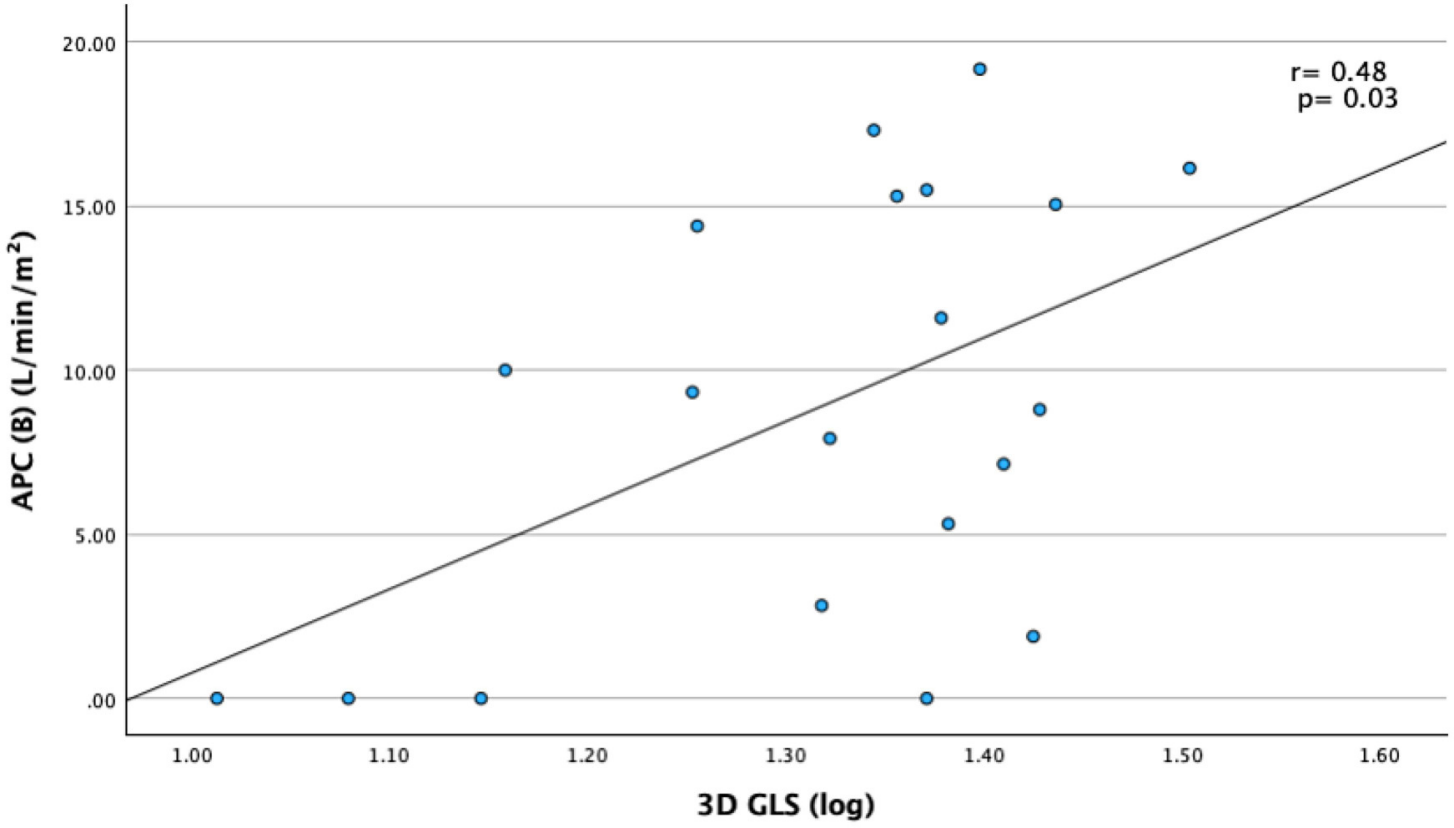
Linear regression illustrating the association between APC-flow calculated using method B as [Ao-(SVC + IVC)] and log-transformed global longitudinal strain measured by 3D echocardiography.

GLS measured by 3D echocardiography was significantly correlated with APC flow calculated using method A (*r* = 0.58, *p* = 0.006) (Figure 7) and method B (*r* = 0.48, *p* = 0.03) (Figure 8). No significant associations were observed between APC flow calculated using either method and EDV or EF% by MRI, nor with EF% or 4CH-longitudinal strain measured by 2D echocardiography (Table 5).

**Table 5:** Correlations between APC calculated with method A and B to MRI- and echocardiographic variables.

Parameter	*p*-value	r
APC (A)—EDV (MRI) (*n* = 27)	0.09	0.33
APC (B)—EDV (MRI) (*n* = 27)	0.165	0.27
APC (A)—EF% (MRI) (*n* = 27)	0.349	0.18
APC (B)—EF% (MRI) (*n* = 27)	0.086	0.33
APC (A)—EF% (3D echo) (*n* = 19)	0.301	0.24
APC (B)—EF% (3D echo) (*n* = 19)	0.329	0.33
APC (A)—4CH-LS (2D echo) (*n* = 26)	0.382	0.175
APC (B)—4CH-LS (2D echo) (*n* = 26)	0.190	0.27

APC, aortopulmonary collaterals, EDV, end-diastolic volume, MRI, magnetic resonance imaging, EF, ejection fraction, 3D echo, three-dimensional echocardiography, 4CH, four chamber, LS, longitudinal strain, 2D echo, two-dimensional echocardiography, GLS, global longitudinal strain.

### Abdominal arterial flow characteristics

Flow in the SMA and CA was evaluated before and after TCPC. In the SMA, peak systolic velocity decreased significantly postoperatively (*p* = 0.015), while end-diastolic velocity increased (*p* = 0.006) and RI decreased (*p* < 0.001). Pre-TCPC SMA RI was significantly correlated with APC flow calculated using method B (*r* = 0.44, *p* = 0.02) and with MRI-derived EF% (*r* = 0.66, *p* = 0.00). No correlation was observed between SMA RI and PE duration or IVC%.

In the CA, no significant changes were observed in flow parameters between pre- and post-TCPC assessments. Although no correlation with APC flow was identified (Method A: *r* = 0.13, *p* = 0.125; Method B: *r* = 0.21, *p* = 0.296), CA RI was significantly associated with EDV measured with MRI (*r* = 0.49, *p* = 0.009).

### Intra- and interobserver variability analysis

The intra-observer analysis of strain analysis demonstrated good agreement for 4CH-longitudinal strain measured by 2D echocardiography (ICC = 0.90) and for EF% measured by 3D echocardiography (ICC = 0.91). In contrast, GLS measured by 3D echocardiography showed moderate agreement (ICC = 0.74). The inter-observer analysis showed good agreement for 4CH-longitudinal strain by 2D echocardiography (ICC = 0.80).

## Discussion

In this prospective study of patients with SV physiology undergoing TCPC, we found that 4CH-longitudinal strain by 2D echocardiography and MRI-derived APC flow were associated with postoperative PE duration. Additionally, GLS measured by 3D echocardiography showed an association with APC flow as calculated by both Method A and Method B. Although the SMA RI was not directly associated with PE duration, it was significantly correlated with APC flow, suggesting a possible link between mesenteric vascular resistance and collateral burden. APC flow calculated as ascending aortic flow minus the sum of superior and inferior vena cava flows (Method B), demonstrated the strongest statistical association with PE duration. Overall, MRI-derived APC flow variables showed stronger statistical associations with PE duration compared with echocardiographic parameters. Lower IVC flow may reflect redistribution of systemic blood flow in the presence of significant APC burden, although it may also represent a marker of more advanced Fontan physiology rather than impaired venous return *per se*. Although several correlations reached statistical significance, the correlation coefficients were modest (*r* ≈ 0.5), indicating only moderate associations. Given the relatively small sample size, these findings should be interpreted cautiously, as statistical significance may reflect sample characteristics rather than strong predictive relationships.

Previous studies have reported mixed findings on the association between APCs and postoperative outcomes, possibly reflecting differences in how collateral flow is quantified. MRI-based assessments have shown the strongest correlations with PE duration and hospital stay (15, 16, 25), while studies relying on angiography or CT have yielded inconsistent results (1, 13, 26, 27). In this study, APC flow was quantified using indirect flow difference methods derived from phase-contrast MRI measurements in major vascular structures. Although direct visualization of individual collateral vessels is often limited by spatial resolution, this indirect approach has been widely used in patients with congenital heart disease to estimate collateral flow (15, 16). MRI flow quantification in young children presents several technical challenges, including high heart rates, partial volume effects, and temporal resolution limitations. These factors may influence the precision of APC flow estimates and should be considered when interpreting the results (28). Despite the growing use of echocardiographic markers such as strain and mesenteric perfusion (17–21), few studies have directly examined their relationship with APC flow or PE duration (25). This gap highlights the need for studies, such as ours, that directly compare MRI and echocardiographic markers in this context.

4CH-longitudinal strain measured by 2D echocardiography was associated with postoperative PE duration. While more negative GLS values typically reflect preserved systolic function, in SV physiology they may indicate increased myocardial deformation due to volume overload from APCs. In SV physiology, interpretation of GLS may differ from that in structurally normal biventricular hearts. More negative GLS values may reflect increased myocardial deformation secondary to volume loading rather than enhanced intrinsic myocardial contractility. Increased preload related to APC flow may therefore result in higher (more negative) GLS values despite preserved or even impaired ventricular efficiency, as suggested by previous studies (10, 29).

In contrast, 3D echocardiographic GLS, EF% and EDV did not correlate with PE duration, possibly due to lower sample size. However, GLS by 3D echocardiography was associated with APC flow using both Method A and B, further supporting the volume overload hypothesis. Unlike 2D echocardiography, which captured deformation from a single apical view and is prone to speckle loss, 3D echocardiography incorporated all myocardial segments, offering more reliable measurements (22). These results suggest that 3D GLS may be a more sensitive marker of volume overload than either 2D echocardiography or measurements of EF% and EDV measured with MRI in this population.

It should also be noted that 3D echocardiographic volumes are systematically underestimated compared with cardiac MRI, which is consistent with prior literature and is also reflected in our dataset (23).

After TCPC, we observed a significant decrease in SMA resistance index and peak systolic velocity, accompanied by an increase in end-diastolic velocity. Although these findings were initially interpreted as reflecting a diastolic runoff or “steal” phenomenon related to APC flow, classic runoff physiology typically manifests as reduced or reversed diastolic flow in downstream arteries. In contrast, the pattern observed in our study (i.e., increased diastolic forward flow with a lower resistance index) is more consistent with reduced downstream vascular resistance. Doppler studies have shown that reductions in peripheral vascular resistance or systemic stroke volume may lead to increased diastolic velocities and decreased resistive indices in the SMA waveform (30). Such Doppler patterns likely reflect mesenteric vasodilation or decreased distal vascular resistance, which may represent an adaptive response aimed at maintaining intestinal perfusion in the setting of altered systemic hemodynamics in single-ventricle circulation (31). Although SMA RI did not correlate directly with pleural effusion duration, its association with MRI-derived APC flow suggests that mesenteric perfusion parameters may still reflect systemic hemodynamic effects related to collateral burden. However, our data do not allow direct confirmation of a steal mechanism, and the observed Doppler changes should therefore be interpreted as reflecting altered mesenteric vascular resistance rather than definitive evidence of diastolic runoff. The association of SMA RI with both APC flow (Method B) and MRI-derived EF% suggests that this Doppler parameter may represent a non-invasive marker associated with APC burden. Flow and RI in the CA did not demonstrate significant changes, which might reflect compensatory flow redistribution within the mesenteric vascular network (18, 32). However, the proposed “venous steal” phenomenon should be interpreted cautiously, as invasive pressure and flow measurements were not available to directly confirm competitive flow dynamics between APC flow and systemic venous return.

To our knowledge, this is the first study to document changes in SMA perfusion in relation to APC burden before and after TCPC. In the present study, mesenteric Doppler measurements were not compared with a separate cohort of healthy controls. Instead, the objective was to assess the relationship between mesenteric perfusion parameters and APC flow as well as postoperative outcomes within the SV population. Therefore, our analysis focused primarily on relative differences within the study population and their association with hemodynamic variables.

We used MRI as the reference for APC quantification, employing two methods: Method A, defined as pulmonary venous return minus pulmonary arterial flow, and Method B, defined as ascending aortic flow minus the sum of SVC and IVC flows (4, 6, 7, 16). APC flow by Method B showed a correlation with PE duration, whereas Method A showed only a non-significant trend. One limitation of Method A is the potential overestimation of APC flow in the presence of systemic-to-pulmonary venous collaterals, which may contribute to pulmonary venous return without passing through the pulmonary arteries, thereby artificially increasing the calculated collateral flow. In contrast, Method B reflects the discrepancy between systemic output and systemic venous return and may therefore provide a more robust estimate of systemic-to-pulmonary collateral flow. Our results align with prior investigations by Glatz et al. (16) and Grosse-Wortmann et al. (9), both of which associate greater collateral burden (by MRI) with longer chest tube durations and hospitalization after TCPC. Our results are congruent with some earlier reports, but contrast with others. Bradley et al. (13) and Zou et al. (1) found no link between APC burden and PE, though those analyses were based on intraoperative or CT-derived assessments, which are less quantitative. McElhinney et al. (26) also reported conflicting outcomes, likely due to their use of angiographic quantification, which relies heavily on contrast dynamics and visual interpretation (13, 26, 33). Triedman et al. (27) highlighted the multifocal origin of APCs, often from subclavian branches, that may be missed by standard aortography unless selective catheterization is used. Such multifocal APCs favor MRI for more reliable quantification. Although MRI itself has limitations, especially when measuring IVC flow near the diaphragm or estimating flow from small or distal feeder vessels (9, 27). As our findings show, outcomes such as PE duration can vary depending on whether APCs are quantified via Method A or Method B, further emphasizing the need for standardization.

We also found an association between lower IVC flow percentage and longer PE drainage duration, suggesting that APC flow may compete with systemic venous return (i.e., venous “steal”). Latus et al. (25) similarly reported reduced IVC flow in patients with greater APC burden. This supports the idea that collateral blood flow competes with systemic venous return, potentially increasing postoperative complications. Moreover, patients with higher CI measured by MRI experienced a longer PE duration. While an elevated CI may arise from volume overload due to APC flow, it may also reflect capillary leak or systemic inflammatory responses that exacerbate pleural fluid accumulation (5).

We did not detect significant associations between PE duration or APC flow and patient age at surgery, ventricular morphology, or cardiopulmonary bypass time. These results mirror findings from Grosse-Wortmann et al. (15) and Mascio et al. (33). Although Gupta et al. (2) found that bypass time correlated with PE volume, it did not predict drainage duration. In contrast, Heinisch et al. (34) identified younger age as a PE risk factor, though their larger patient cohort size may have enabled detection of effects not evident in smaller cohorts.

Our findings support an association between APC burden and both pulmonary and systemic flow alterations and highlight the complementary role of echocardiography in this context. While MRI remains the reference standard for quantifying APC flow, echocardiographic parameters such as GLS and SMA Doppler indices appear to capture its downstream hemodynamic consequences, including ventricular loading and mesenteric perfusion changes. Given its bedside availability and feasibility for serial assessment, echocardiography may provide a practical, non-invasive tool for functional evaluation in the pre-TCPC setting. Rather than directly measuring collateral flow, these parameters likely reflect the integrated systemic response to APC burden and may therefore assist in identifying patients with unfavorable hemodynamic profiles who could benefit from closer perioperative monitoring and tailored management strategies.

## Limitations

Like many pediatric studies in congenital heart disease, our study was limited by a relatively small sample size and incomplete imaging datasets, precluding multivariable analyses. As a result, the observed associations may reflect shared hemodynamic influences rather than independent predictors. The heterogeneous cohort and inability to stratify by diagnosis or ventricular morphology may have further masked subgroup-specific effects. The wide range in PE duration, may reflect additional unmeasured risk factors such as lymphatic dysfunction, postoperative management differences, or inflammatory responses, which were not systematically assessed in this study. Image quality constraints, particularly in 3D echocardiography and MRI, led to exclusion of several datasets and limited completeness of analysis. Acquisition of high-quality 3D datasets in young children is technically challenging due to high heart rates, motion, and limitations of ECG-gated multi-beat imaging, increasing the risk of stitching and tracking artefacts (23). Invasive validation of APC flow was not performed, and MRI flow analysis was conducted by a single non-blinded observer without formal assessment of inter- or intra-observer variability, which may introduce measurement bias. Postoperative outcomes may also have been influenced by clinical management factors, as chest tube removal was not protocolized, and data on pleural effusion volume, postoperative medications, and other contributors such as pulmonary artery anatomy or diaphragmatic function were not available. Mesenteric resistance indices are influenced by multiple physiological factors that were not controlled for in this study. Several established determinants of prolonged pleural effusion after TCPC, such as pulmonary vascular resistance, Fontan pressure, ventricular diastolic function, atrioventricular valve regurgitation, lymphatic abnormalities, and nutritional status, were not systematically assessed. Therefore, the observed associations between APC flow, GLS, SMA resistance index, and pleural effusion duration may partly reflect overall hemodynamic severity of single-ventricle physiology rather than a specific causal relationship and should be interpreted as exploratory rather than predictive or mechanistic.

Despite these limitations, the prospective design and comprehensive multimodality imaging approach provide clinically relevant insights into the relationship between APC burden and postoperative outcomes.

## Conclusion

Echocardiographic markers of myocardial deformation and mesenteric perfusion were associated with MRI-derived APC flow and postoperative pleural effusion duration, suggesting that these non-invasive parameters reflect the hemodynamic impact of collateral burden in patients undergoing TCPC. While MRI remains the reference method for APC quantification, echocardiography may provide complementary functional information on ventricular loading and systemic perfusion. Given the exploratory nature of this study, larger and more standardized investigations are required to confirm these associations and to define the potential role of echocardiographic assessment in preoperative risk stratification.

## Data Availability

The raw data supporting the conclusions of this article will be made available by the authors, without undue reservation.

## References

[1] Zou M Wang Y Cui H Ma L Yang S Xia Y. Outcomes of total cavopulmonary connection for single ventricle palliation. J Thorac Dis. (2016) 8(1):43–51. 10.3978/j.issn.2072-1439.2016.01.41 26904211 PMC4740135

[2] Gupta A Daggett C Behera S Ferraro M Wells W Starnes V. Risk factors for persistent pleural effusions after the extracardiac Fontan procedure. J Thorac Cardiovasc Surg. (2004) 127(6):1664–9. 10.1016/j.jtcvs.2003.09.011 15173721

[3] Nabeshima T Ishikawa Y Sumitomo N Go K Kodama Y Kuraoka A. The impact of the pulmonary artery Index and aortopulmonary collateral artery coil embolization on intractable pleural effusions after a Fontan surgery. Int Heart J. (2021) 62(3):559–65. 10.1536/ihj.20-498 33994500

[4] Grosse-Wortmann L Al-Otay A Yoo SJ. Aortopulmonary collaterals after bidirectional cavopulmonary connection or fontan completion: quantification with MRI. Circ Cardiovasc Imaging. (2009) 2(3):219–25. 10.1161/CIRCIMAGING.108.834192 19808596

[5] Schmiel M Kido T Georgiev S Burri M Heinisch PP Vodiskar J. Aortopulmonary collaterals in single ventricle: incidence, associated factors and clinical significance. Interact Cardiovasc Thorac Surg. (2022) 35(2):ivac190. 10.1093/icvts/ivac190 35876534 PMC9318886

[6] Odenwald T Quail MA Giardini A Khambadkone S Hughes M Tann O. Systemic to pulmonary collateral blood flow influences early outcomes following the total cavopulmonary connection. Heart. (2012) 98(12):934–40. 10.1136/heartjnl-2011-301599 22626901

[7] Whitehead KK Harris MA Glatz AC Gillespie MJ DiMaria MV Harrison NE. Status of systemic to pulmonary arterial collateral flow after the fontan procedure. Am J Cardiol. (2015) 115(12):1739–45. 10.1016/j.amjcard.2015.03.022 25907503 PMC4450112

[8] Mohammad Nijres B Aregullin EO Al-Khatib Y Samuel BP Abdulla R-i Hijazi ZM. Aortopulmonary collaterals in single ventricle physiology: variation in understanding occlusion practice among interventional cardiologists. Pediatr Cardiol. (2020) 41(8):1608–16. 10.1007/s00246-020-02418-8 32720087

[9] Grosse-Wortmann L Hamilton R Yoo SJ. Massive systemic-to-pulmonary collateral arteries in the setting of a cavopulmonary shunt and pulmonary venous stenosis. Cardiol Young. (2007) 17(5):548–50. 10.1017/S1047951107000960 17640400

[10] Mkrtchyan N Frank Y Steinlechner E Calavrezos L Meierhofer C Hager A. Aortopulmonary collateral flow quantification by MR at rest and during continuous submaximal exercise in patients with total cavopulmonary connection. J Magn Reson Imaging. (2018) 47(6):1509–16. 10.1002/jmri.25889 29105891

[11] Kitano M Yazaki S Kagisaki K. Aggressive coil embolization for connected aortopulmonary collateral arteries with large shunts developed after diaphragmatic plication performed after cavopulmonary connection to facilitate Fontan circulation. Catheter Cardiovasc Interv. (2013) 82(5):E694–703. 10.1002/ccd.25094 23804520

[12] Banka P Sleeper LA Atz AM Cowley CG Gallagher D Gillespie MJ. Practice variability and outcomes of coil embolization of aortopulmonary collaterals before Fontan completion: a report from the pediatric heart network fontan cross-sectional study. Am Heart J. (2011) 162(1):125–30. 10.1016/j.ahj.2011.03.021 21742098 PMC3137245

[13] Bradley SM McCall MM Sistino JJ Radtke WAK. Aortopulmonary collateral flow in the Fontan patient: does it matter? Ann Thorac Surg. (2001) 72(2):408–15. 10.1016/S0003-4975(01)02813-2 11515875

[14] Ridderbos F-JS Chan FP van Melle JP Ebels T Feinstein JA Berger RMF. Quantification of systemic-to-pulmonary collateral flow in univentricular physiology with 4D flow MRI. Cardiol Young. (2023) 33(9):1634–42. 10.1017/S1047951122002840 36120930

[15] Grosse-Wortmann L Drolet C Dragulescu A Kotani Y Chaturvedi R Lee K-J. Aortopulmonary collateral flow volume affects early postoperative outcome after Fontan completion: a multimodality study. J Thorac Cardiovasc Surg. (2012) 144(6):1329–36. 10.1016/j.jtcvs.2012.03.032 22502974

[16] Glatz AC Rome JJ Small AJ Gillespie MJ Dori Y Harris MA. Systemic-to-pulmonary collateral flow, as measured by cardiac magnetic resonance imaging, is associated with acute post-fontan clinical outcomes. Circ Cardiovasc Imaging. (2012) 5(2):218–25. 10.1161/CIRCIMAGING.111.966986 22228054 PMC3310971

[17] Taylor GA. Blood flow in the superior mesenteric artery: estimation with Doppler US. Radiology. (1990) 174(1):15–6. 10.1148/radiology.174.1.2403677 2403677

[18] Johnson JN Ansong AK Li JS Xu M Gorentz J Hehir DA. Celiac artery flow pattern in infants with single right ventricle following the Norwood procedure with a modified Blalock-Taussig or right ventricle to pulmonary artery shunt. Pediatr Cardiol. (2011) 32(4):479–86. 10.1007/s00246-011-9906-y 21331516 PMC3139997

[19] Carlo WF Kimball TR Michelfelder EC Border WL. Persistent diastolic flow reversal in abdominal aortic Doppler-flow profiles is associated with an increased risk of necrotizing enterocolitis in term infants with congenital heart disease. Pediatrics. (2007) 119(2):330–5. 10.1542/peds.2006-2640 17272623

[20] del Castillo SL Moromisato DY Dorey F Ludwick J Starnes VA Wells WJ. Mesenteric blood flow velocities in the newborn with single-ventricle physiology: modified Blalock-Taussig shunt versus right ventricle-pulmonary artery conduit. Pediatr Crit Care Med. (2006) 7(2):132–7. 10.1097/01.PCC.0000200999.89777.92 16474253

[21] Harrison AM Davis S Reid JR Morrison SC Arrigain S Connor JT. Neonates with hypoplastic left heart syndrome have ultrasound evidence of abnormal superior mesenteric artery perfusion before and after modified Norwood procedure. Pediatr Crit Care Med. (2005) 6(4):445–7. 10.1097/01.PCC.0000163674.53466.CA 15982432

[22] Sato T Calderon RJ Klas B Pedrizzetti G Banerjee A. Simultaneous volumetric and functional assessment of the right ventricle in hypoplastic left heart syndrome after fontan palliation, utilizing 3-dimensional speckle-tracking echocardiography. Circ J. (2020) 84(2):235–44. 10.1253/circj.CJ-19-0926 31932561

[23] Muraru D Niero A Rodriguez-Zanella H Cherata D Badano L. Three-dimensional speckle-tracking echocardiography: benefits and limitations of integrating myocardial mechanics with three-dimensional imaging. Cardiovasc Diagn Ther. (2018) 8(1):101–17. 10.21037/cdt.2017.06.01 29541615 PMC5835646

[24] Heiberg E Sjögren J Ugander M Carlsson M Engblom H Arheden H. Design and validation of segment–freely available software for cardiovascular image analysis. BMC Med Imaging. (2010) 10:1. 10.1186/1471-2342-10-1 20064248 PMC2822815

[25] Latus H Gummel K Diederichs T Bauer A Rupp S Kerst G. Aortopulmonary collateral flow is related to pulmonary artery size and affects ventricular dimensions in patients after the fontan procedure. PLoS One. (2013) 8(11):e81684. 10.1371/journal.pone.0081684 24303064 PMC3841134

[26] McElhinney DB Reddy VM Tworetzky W Petrossian E Hanley FL Moore P. Incidence and implications of systemic to pulmonary collaterals after bidirectional cavopulmonary anastomosis. Ann Thorac Surg. (2000) 69(4):1222–8. 10.1016/S0003-4975(99)01088-7 10800823

[27] Triedman JK Bridges ND Mayer JE Lock JE. Prevalence and risk factors for aortopulmonary collateral vessels after fontan and bidirectional glenn procedures. J Am Coll Cardiol. (1993) 22(1):207–15. 10.1016/0735-1097(93)90836-P 8509543

[28] Weinberg PM Fogel MA. Cardiac MR imaging in congenital heart disease. Cardiol Clin. (1998) 16(2):315–48. 10.1016/S0733-8651(05)70015-1 9627763

[29] Luo Q Zhao W Su Z Liu Y Jia Y Zhang L. Risk factors for prolonged pleural effusion following total cavopulmonary connection surgery: 9 Years’ experience at Fuwai hospital. Front Pediatr. (2019) 7:456. 10.3389/fped.2019.00456 31788459 PMC6854004

[30] Perko MJ Perko G Just S Secher NH Schroeder TV. Changes in superior mesenteric artery Doppler waveform during reduction of cardiac stroke volume and hypotension. Ultrasound Med Biol. (1996) 22(1):11–8. 10.1016/0301-5629(95)02037-3 8928308

[31] Taourel P Perney P Dauzat M Gallix B Pradel J Blanc F. Doppler Study of fasting and postprandial resistance indices in the superior mesenteric artery in healthy subjects and patients with cirrhosis. J Clin Ultrasound. (1998) 26(3):131–6. 10.1002/(sici)1097-0096(199803/04)26:3<131::aid-jcu4>3.0.co;2-n 9502035

[32] van Petersen AS Kolkman JJ Meerwaldt R Huisman AB van der Palen J Zeebregts CJ. Mesenteric stenosis, collaterals, and compensatory blood flow. J Vasc Surg. (2014) 60(1):111–119.e1-2. 10.1016/j.jvs.2014.01.063 24650741

[33] Mascio CE Austin EH III. Pleural effusions following the Fontan procedure. Curr Opin Pulm Med. (2010) 16(4):362–6. 10.1097/MCP.0b013e3283396efc 20410822

[34] Heinisch PP Metz P Staehler H Mayr B Vodiskar J Strbad M. Pleural and mediastinal effusions after the extracardiac total cavopulmonary connection: risk factors and impact on outcome. Front Cardiovasc Med. (2022) 9:1026445. 10.3389/fcvm.2022.1026445 36426216 PMC9678908

